# An Address, Read before the Madison County Medical Society November, 1854

**Published:** 1854-12

**Authors:** John S. Dewey

**Affiliations:** Troy, Ill.


ART. II.—*An Address*, read before the Madison county Medical Society, November, 1854. By JOHN S. DEWEY, M.D.

GENTLEMEN,—In accordance with a regulation of this Society, requiring a contribution from each member for our mutual interest or benefit, I purpose to communicate the deductions relative to the disease called "Milk Sickness," which a number of years' practice in a locality where it has, from the first settlement of the country until the present time, been peculiarly fatal to the inhabitants, and to such of their domestic animals as are obnoxious to it, has enabled me to draw.

The greatest diversity of opinion prevails on the subject of this disease among numbers of the profession, owing to the want of the means of information. Our text-books do not admit it into the catalogue of human maladies; our medical journals seldom contain even the most meagre expression of opinion of it; and, as the cause which produces it is happily confined to circumscribed and widely separated localities, comparatively few practitioners ever have an opportunity of witnessing its phenomena. Therefore there are intelligent physicians who even doubt the existence of this disease; which has been witnessed or treated by a large number of us. According to the rules of evidence, we can establish the fact that such a disease, sui generis, does exist; by our testimony that we have seen it, *smelt it*, and anxiously watched its singular phenomena, though all the profession beside ignore it.

Its name is furnished us by the uninitiated in plain English, and is not inappropriate; for the virus is usually communicated to the human system, and brought to our notice through the medium of milk. The flesh of the infected animal seldom contains the poison sufficiently concentrated to impart its effect by the quantity usually eaten.

The name may also refer to the affinity which the poison manifests for milk. It invariably runs harmlessly through the system of the animal in which this secretion is active; but shows its virulence on the offspring or individual, which takes the secretion into the stomach.

If we prefer the language usually employed in our nomenclature, we have a name sufficiently euphonic and descriptive, and which I see no good reason to exchange for any other—morbus lactealis, or morbus ab lactæ.

Various theories prevail with regard to the cause of the disease: some attribute it to a malaria which arises from the earth in certain damp and fertile localities; others, to a mineral, the sediment of which may sometimes be seen in stagnant pools, when there is a general scarcity of water; and, fortunately for this theory, cattle are driven to these places at the very season of the year when milk-sickness is most prevalent—some, to the web of an insect; and others, to a succulent plant, which grows in damp and thickly-shaded bottoms, and bears a white blossom until late in autumn, and which, if not really guilty of the evils attributed to it, is, at any rate, from its habits and association, justly and very generally suspected.

I shall not attempt to refute the various arguments used to prove, that among the first named articles, is to be found the insidious poison; but will briefly state the reasons which have convinced my mind, that it will yet be detected, and demonstrated, in the last named plant. 1st. It is invariably found in this State; in Ohio and Kentucky; whenever milksickness is endemic; and in our own county, it is demonstrable, that it flourishes most abundantly in the immediate locations where the disease is most rife. In the latter part of this summer, a friend of mine from Washington county in this State remarked, that he had not seen this plant on his journey to Jacksonville, except in a bottom about a mile south of Edwardsville; and unhesitatingly averred that *there* was Milk-sickness. We know the truth of his conclusion, however we may condemn his premises. He emigrated from a portion of Ohio where this disease prevails, and where this plant also flourishes. 2nd. I know that all the symptoms of the disease were produced on calves, at two several times, by feeding them with this plant for experiment. 3rd. Whenever Milksickness is endemic, the plant can invariably be found in the vicinity. And during the past unusual season, when the parched scanty vegetation of uplands on our south, drove the cattle from their usual haunts, to the bottoms and ravines, where this suspected plant is known to grow; the disease has been brought to localities where it was before unknown.

This plant is sought with avidity by cattle late in summer, when other vegetation is matured and dry, because it is then in bloom and more succulent. The same peculiarity renders it more easily destroyed by the first hard frost of autumn, when it and the disease cotemporaneously disappear.

Much more concurrent evidence might be adduced, fortifying the same conclusion, and defacing the theories ingeniously framed to prove the mineral or malarial origin of the poison; such as the removing it from certain localities where it was known to exist, by clearing and fencing the lands for pasture. But I abstain from further speculation on this subject; believing from the interest now manifest in the subject, that we will soon be able to point with absolute certainty to the mysterious agent of so much affliction to certain portions of the west.

I intended to have learned to what class in botany this plant belongs before this meeting, and to have tried some other experi ments with it, but have been prevented by procrastination.

The symptoms of Milksickness at the outset, somewhat resembles those of our autumnal fevers, with congestion of the stomach. In both there is fever, languor, pain in the back and limbs, with a depressed and feeble pulse, but the similarity extends no further; and if we still persist in classing it among the miasmatic fevers, we are bewildered and annoyed by the inconsistency of the subsequent symptoms.

In the latter, the tongue is pointed and its edges red. In the former we are surprised, with so much gastric irritation, to find it generally relaxed, the villi long, and covered with a white coat, and the edges pallid.

In the latter, the patient complains of a sense of heat and pain in the stomach, which is relieved in some degree by the administration of an anodyne, emollient drinks and counter irritation.

In the former, the distress is more a sense of fulness and oppression, from which little or no relief is afforded by the treatment above; and the practitioner is urged by the sufferer to administer an emetic to remove the imagined accumulation.

In milk-sickness there is a fetor emanated, which is truly pathognominic. It pervades the apartment where the patient is confined; is readily observed by every by-stander; and, when once perceived, is not soon forgotten. It is familiar to the inhabitant of afflicted districts; and when they detect it in a sick room they unhesitatingly pronounce their diagnosis; and no argument or authority adduced by the medical attendant can induce them to change it, or in the least shake their confidence in its correctness. Indeed, their arguments in support of their conclusion would often confound the most learned and sceptical of our profession.

They tell us of our cattle (except their milch cows) affecte with trembles, and evident enervation, and having this peculiar fitor, and will often show you a calf in the place where your patient is which had partaken of milk from the same cow, with similar symptoms of enervation and the same odor; or if the calf shows no indisposition, the disease will be developed by actually exercising him about the yard. This I have more than once witnessed, and I cannot believe that conjestive fever, with the peculiar odor, trembles, and enervation of which I have spoken, could be produced for the occasion by exercise. Or they may refer you to their carniverous animals, that have eaten of the carcasses of beasts dead of this disease, manifesting the same symptoms and odor.

In autumnal fevers complicated with congestion of the stomach the breathing may be normal, or slightly hurried and short. In milksickness it is labored, deep, and difficult, and at times performed as if by a dispairing effort of the will.

The bowels are most obstinately constipated. The surface is moist, and grows more and more cool as the circulation languishes; and unless relieved by proper treatment, the patient either dies from asthenia or remains for years an invalid, subject to a recurrence of all the symptoms. from fatigue or violent exercise.

From these facts I am led to infer, that the poison acts not upon the mucous membrane of the stomach as an irritant to excite inflammation of the organ ; but upon the nervous system, in such a manner as partially to paralyze the power of muscular contractibility.

We see this influence in the voluntary muscles, which immediately on the attack, refuse to sustain the weight of the patient, and tremble as if outstrained in the effort. The same condition may prevail with the heart, diminishing the force of its contractions, and with the middle coat of the bowels suspending the peristaltic action, thus producing the obstinate constipation, which is a prominent and very difficult symptom in the disease, and on which possibly depends, the sense of fulness in the stomach, and constant retching.

That the motor portion of the nervous system is susceptible to such an impression as I have here claimed for the poison producing milksickness, is proven by well established facts, showing, that certain substances do act specifically upon separate portions of the nervous system; as digitalis in diminishing, or *upas antiar* in suspending the action of of the heart; or, as the analogous effect of lead, in painter's cholic.

This view of the pathology will explain more of the symptoms, than any other theory which I am aware of having been advocated and is very satisfactionally corroborated by the post-mortem appearances of the viscera.

When death occurs within the first few days from the attack, I am credibly informed, there is no trace of inflammatory action, or local lesion whatever. I have never been permitted to examine the human body, but in the carcasses of animals dead of this disease, which I have examined minutely, the only abnormal appearance which I have observed with any degree of regularity was, the utter siccity of the fecal contents of the bowels. I have seen it in the ox so perfectly destitute of moisture that it would readily ignite.

The gall bladder contains healthy bile, often in large quantity; and is not emptied into the duodenum, owing to the same condition of the muscular fibre of the cist and duct, which holds with the bowels, and prevents their contents from being expelled; but why the absorbents should continue to take up the fluid in the alimentary canal, when the function of the exhalents is suspended, I shall not attempt to explain.*

We are also referred to the nervous system for the pathology of the disease; by the recurrence of the symptoms, from exercise or fatigue, without the reapplication of the cause which originally produced them.

Of the treatment I have but little to say. With our present knowledge, we have no antidote to neutralize the poison, or substance which will counteract its depressing influence upon the nervous system; and our only alternative is, to ward off as well as may be, its effect upon certain organs when the danger is most imminent, until the instinctive healing power in the system shall come to our aid, and expel the poison by the emunctories, or persuade the restive nervous system to tolerate its stay.

The first indication then is to overcome the constipation of the bowels. By this we expect to accomplish a double purpose for good; 1st, to relieve the stomach, upon the same principle that it is relieved by reducing strangulated hernia, or removing any other obstruction to their action; and 2nd, to open an outlet for the offending matter.

In the first we are generally pleased with the result. I have often known the stomach relieved immediately on the contact of the purgative stimulants, with the extremities of the nerves of the bowels, encouraging their action.

The stomach does not usually complain while the bowels are free.

The purgative which I prefer is the proto chloride of mercury, in 20, 30, or 60 gr. doses; given in mucilage of gum arabic; because its specific gravity makes it retained, while the retching would reject almost any other substance. The only caution necessary in the use of this article is, to be sure to give enough; so that our object may be accomplished with certainty, without danger of producing ptyalism, from which I have never known any good result.

After acting freely upon the bowels, if there are periodical exa-

* Might not the absorbents act under the hydraulic law, by capillary attraction and the vital function of the exhalents be suspended?

cerbations and remissions in the symptoms, I give sulphate of quinine in the remissions; but if this periodicity does not appear as is most frequently the case, I am driven to the employment of mere placebos or innocent and as yet fruitless experiments, until the condition of the bowels require another purgation; when I repeat the pro. chlo. of mercury in doses as before.

Though the matter ejected from the stomach usually shows an acid reaction, I have never afforded any relief by the use of either, alkalies, or vegetable or mineral acids.

I use vesication over the epegastrium; but with little apparent benefit.

The sheet anchor of my hope in the treatment of this disease is purgation, and the friendly interposition of the vis medicatrix natura.

Thus have I briefly and imperfectly communicated the deductions from my observation and experience in the disease called Milksickness, which we are more frequently called to tr at and therefore have better opportunities to investigate, than our brethren in almost any other locality; and for this reason it becomes peculiarly our duty, to our profession and humanity to ferret out its cause, its true pathology and a proper plan of treatment.

Troy, Ill., Nov. 31st, 1854.

